# Fear of movement in patients after lumbar spine fusion and an analysis of factors: a cross-sectional study

**DOI:** 10.3389/fneur.2025.1467843

**Published:** 2025-03-27

**Authors:** Yingyan Pan, Qiong Qi, Chao Yang, Meng Dai, Huihui Zhang, Jie Wen, Hailing Qiu

**Affiliations:** ^1^Department of Orthopedics, Hunan Provincial People's Hospital, The First Affiliated Hospital of Hunan Normal University (Hunan Provincial People’s Hospital), Changsha, China; ^2^The First Affiliated Hospital of Hunan Normal University (Hunan Provincial People’s Hospital), Changsha, China

**Keywords:** kinesiophobia, lumbar interbody fusion, low back pain, rehabilitation, cross-sectional study

## Abstract

**Background:**

Numerous studies have confirmed the significant benefits of exercise rehabilitation in both preoperative and postoperative treatment of lumbar disc herniation. However, there is a prevalent fear or avoidance of exercise among patients with chronic low back pain prior to surgery, while research on exercise fear after lumbar fusion remains insufficient. This study aims to investigate the incidence and severity of exercise fear in patients with chronic low back pain and leg pain following lumbar fusion surgery, as well as analyze its underlying mechanism and associated risk factors.

**Methods:**

A cross-sectional study was conducted on patients undergoing posterior lumbar fusion for lumbar disc herniation between May 2023 and January 2024. The Tampa Motor Phobia Scale (TSK-17) was utilized to assess motor fear among participants. Additionally, clinical and imaging risk factors were analyzed through multivariate regression analysis to determine relevant influencing factors.

**Results:**

Following strict inclusion and exclusion criteria, a total of 178 patients who underwent posterior lumbar fusion were included in this study, comprising 104 males (58.4%). Kinesiophobia was defined as a TSK-17 score ≥ 37, which identified 65.2% (116/178) of the screened patients exhibiting motor phobia. Multivariate regression analysis revealed that motor phobia was strongly associated with age, higher levels of pain intensity, elevated Beck Depression Inventory (BDI) scores, lower General Self-Efficacy Scale (GSES) scores, increased number of surgical levels involved during operation, greater amount of postoperative incision drainage, higher degree of nerve root compression observed on preoperative lumbar MRI scans, as well as smaller area occupied by the paravertebral muscles in the lumbar region.

**Conclusion:**

This study has identified a significantly high incidence of postoperative exercise fear in patients undergoing posterior lumbar fusion, along with potential risk factors. Therefore, it is crucial for clinicians to closely evaluate and monitor these patients in order to develop appropriate strategies for postoperative exercise rehabilitation.

## Introduction

Lumbar interbody fusion (LIF) is a primary surgical technique for treating Lumbar disc herniation (LDH), which has demonstrated significant efficacy and widespread clinical application (1). LIF surgery effectively alleviates clinical symptoms, prevents neurological function deterioration by fully decompressing the dural sac and nerve roots compressed by the intervertebral disc’s nucleus pulposus, and achieves stable internal fixation and reliable fusion (2, 3). With an increasing incidence of LDH in younger individuals and an aging population, there has been a steady rise in the number of LIF surgeries performed annually, leading to a growing demand for postoperative rehabilitation (4, 5). Numerous studies have confirmed that exercise rehabilitation plays a crucial role in both pre- and post-operative treatment of lumbar disc herniation (6). Postoperative exercise rehabilitation is an essential component of non-pharmacological therapy aimed at enhancing lower back muscle strength, improving lumbar spine stability, reducing postoperative pain, enhancing lumbar function recovery, and ultimately improving patients’ quality of life (7–9). Typically initiated upon patient awakening from anesthesia after lumbar surgery with focus on the first six months following surgery. Early exercises primarily involve axis turning as well as sitting-to-standing transitions training. Subsequently progressing to standing up from bed followed by walking exercises. Other therapeutic exercises include early ankle pump exercises along with single straight leg raises while double straight leg raises are introduced during intermediate stages. Later stages incorporate yoga ball training into the program (10).

Kinesiophobia refers to an excessive, irrational fear and avoidance of movement or activity that can be debilitating (11). During the acute stage of pain, fear of exercise serves as a defensive behavior strategy to protect the body from further injury (12). However, in the long term, it becomes detrimental as it may lead to decreased motor function and disability due to lack of physical activity, while also increasing the risk of depression and anxiety (13). Research has demonstrated a positive correlation between the severity of kinesiophobia and postoperative pain intensity and dysfunction, while negatively impacting quality of life (13, 14).

Kinesiophobia is prevalent in the preoperative phase of patients with chronic Low Back Pain (LBP) (15). Even after surgical intervention to relieve pathological compression, a significant number of patients continue to exhibit preoperative exercise psychology and behavior (16, 17). A study (12) revealed that LBP patients with high levels of exercise phobia had a 41% higher risk of physical disability compared to those without exercise phobia. Given that Lumbar Interbody Fusion (LIF) entails longer duration and greater trauma than simple discectomy or decompression, it results in worse lower back muscle strength and increased damage, making early motor rehabilitation particularly crucial (18). Currently, there is insufficient research on exercise fear among patients following LIF surgery, with most studies limited to investigating patient compliance with exercise rehabilitation training. Previous studies have identified kinesiophobia as an important factor influencing compliance with exercise rehabilitation in chronic LBP patients; however, few studies have explored the occurrence of kinesiophobia specifically in post-LIF surgery patients. Furthermore, existing studies primarily analyze the relationship between exercise fear and subjective variables such as pain, self-care ability, social support, and emotion while neglecting objective imaging variables. The decline in function observed in key core muscles like multifidus and erector spinae is closely associated with LBP (19). Fear of exercising can exacerbate low back pain symptoms after surgery; nevertheless, whether there exists a causal relationship between lumbar and dorsal muscles’ condition and post-surgery fear of exercising remains unexplored.

Therefore, the objective of this study is to comprehensively investigate the prevalence of kinesiophobia in patients following lumbar interbody fusion (LIF) by considering demographic characteristics, general clinical data, and imaging parameters. Additionally, we aim to analyze the factors influencing kinesiophobia and provide guidance for healthcare professionals to promptly implement intervention measures such as health education and exercise rehabilitation programs for high-risk individuals. Ultimately, our goal is to prevent delayed recovery or dysfunction of the lumbar spine caused by exercise phobia.

## Methods

### Study population

A cross-sectional study was conducted on patients with lumbar disc herniation who underwent posterior lumbar fusion surgery at our orthopedic center between May 2023 and January 2024. This study received ethical approval from our hospital’s Ethics Committee (Approval number: 2022-119), and all participants were provided with informed consent before voluntarily participating in the study.

The inclusion criteria for this study were as follows: (1) Lumbar disc herniation was diagnosed based on clinical symptoms, with a disease duration of more than 3 months, regardless of the presence or absence of lumbar spinal stenosis; (2) Pathological anatomical features corresponding to clinical symptoms were confirmed by clear and accurate MRI imaging; (3) Surgical indications for posterior lumbar interbody fusion were met: A history of lumbar intervertebral disc protrusion for more than 6 to 12 weeks, with no effect after systematic conservative treatment; or symptoms worsen or recur during conservative treatment; or severe pain, or the patient is in a forced position, affecting work or life; or single nerve paralysis or cauda equina nerve paralysis occurs, manifested as muscle paralysis or rectal and bladder symptoms; (4) Participants aged between 18 and 70 years old were included.

Exclusion criteria: (1) Cognitive impairment, disturbance of consciousness, or mental disorder will be grounds for exclusion; (2) Refusal to participate in the study will result in exclusion; (3) Communication disorders will lead to exclusion; (4) Inability to complete the planned operation due to any reason will be considered as an exclusion criterion; (5) Coexistence of other acute traumas such as spinal fracture and hip fracture will result in exclusion; (6) Lower limb muscle strength less than grade 3 before operation and less than grade 4 after operation will serve as an exclusion criterion; (7) Previous history of spinal surgery is a ground for exclusion; (8) Presence of severe osteoarthritis, infection, tuberculosis, malignant tumor, heart failure, pulmonary dysfunction, muscle atrophy or any medical condition that may interfere with exercise are all reasons for potential exclusions.

### Data collection and outcome measures

We extracted comprehensive clinical data from the patient’s electronic medical record system, encompassing gender, age, height, weight, marital status, education level, disease duration, occupation (manual or mental worker), primary caregiver information, operative segment details, number of postoperative wound drainage tubes employed, total postoperative wound drainage volume recorded, current analgesic usage patterns and comorbidities (Charlson comorbidity index score was calculated). Additionally collected were factors potentially influencing motor and neurologic recovery such as alcohol and smoking history pre- and post-hospitalization.

The clinical outcome data encompassed pain experienced during exercise, the onset of exercise-induced pain, and the Oswestry Disability Index (ODI) (20). Pain intensity was assessed using a visual analogue scale (VAS, 1–10). Subjective emotional factors that could potentially impact postoperative exercise were evaluated through the use of the General Self Efficacy Scale (GSES) (21) and Beck Depression Inventory (BDI-13) (22).

### Assessment of kinesiophobia

The Tampa Scale of Kinesiophobia (TSK-17) (23) was utilized to evaluate the presence of kinesiophobia, a self-reported questionnaire comprising 17 items categorized into four response options: strongly agree, agree, disagree, and strongly disagree. Each item was scored on a scale ranging from 1 to 4. Items 4, 8, 12, and 16 were reverse-scored. Total scores ranged from 17 to 68 with higher scores indicating greater severity of kinesiophobia. The TSK-17 is widely recognized as a reliable measure for assessing kinesiophobia (24, 25). A total TSK-17 score equal to or exceeding 37 indicates the presence of kinesiophobia. The Chinese version of TSK-17 (26) demonstrated strong internal consistency (Cronbach’s *α* coefficient = 0.82) and test–retest reliability (intraclass correlation coefficient ICC = 0.90). In this study, patients were classified into two groups based on their TSK-17 scores: the kinesiophobia group (TSK-17 score ≥ 37) and the non-kinesiophobia group (TSK-17 score ≤ 36).

### Radiology assessment

The patient’s preoperative lumbar spine MRI was acquired from the hospital imaging system HIS for the assessment of nerve root compression and lumbar paravertebral core muscle area in the patient.

Grading of nerve root compression was assessed based on the research criteria established by Pfirrman et al. (27). The degree of nerve root compression was evaluated using MRI cross-sectional imaging of the segment with the most severe lumbar disc herniation, which was categorized into four grades ranging from 0 to III: grade 0 (normal) indicated no evident contact between the disc and the nerve root; Grade I (contact) denoted that although the disc was adjacent to the nerve root, there were no signs of deviation or deformation in the nerve root; Grade II (offset) indicated displacement of the nerve root due to disc compression; Grade III (compression) referred to flattening of the nerve root caused by compression from both nucleus pulposus and vertebral canal wall. Compared with measuring the sagittal diameter index (SI) (28, 29), this method is more intuitive and convenient to evaluate the degree of nerve root compression.

**Figure 1:**
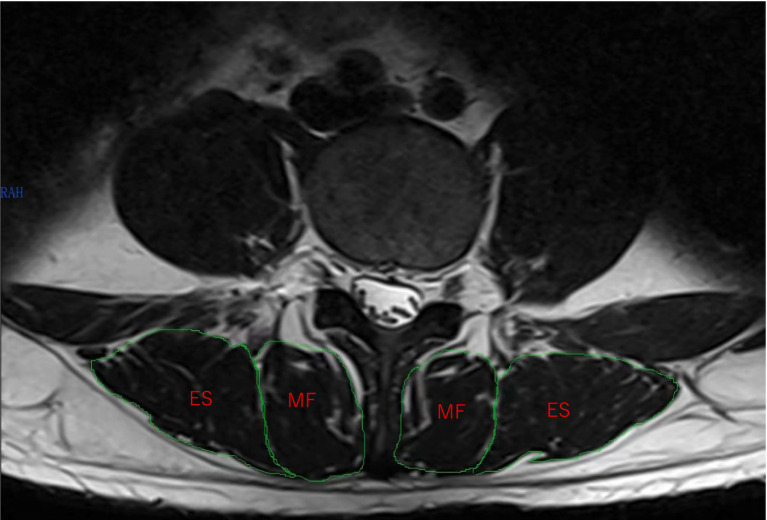
Cross-sectional magnetic resonance imaging of the fourth lumbar spine. MF, multifidus; ES, erector spinae.

Lumbar core muscle area (bilateral multifidus and erector spinae) data were collected. Prior to the operation, all patients underwent a lumbar MRI plain scan using a Siemens 3.0 T MRI imager with T2 sequence parameters: repeat time of 3,500 ms, echo time of 94 ms, and a scan matrix of 256 × 51. The MRI scanning axis was kept parallel to the endplate of the lumbar spine, and fifteen images were scanned in axial position (3 images of the upper, central and lower part of each intervertebral space). In this study, the morphology and structure of the multifidus and erector spinae muscles at the central level of the L4/5 intervertebral space were measured on bilateral cross sections (Figure 1). Compared with the images of the upper and lower layers of the intervertebral space, the images of the central level of the intervertebral space avoided the vertebral body, and the muscle area imaging was fuller and clearer. To minimize potential bias caused by different body positions, patients were positioned in a prone position during the MRI examination. All imaging measurements were performed by an investigator who was not involved in patient treatment.

### Statistical analysis

The statistical analysis was performed using SPSS 25.0 software. Continuous variables with a normal distribution were described as mean ± standard deviation (X ± S) and compared using an independent t-test. Non-normally distributed continuous variables were described as median (quartile) [M (P25, P75)], and group comparisons were analyzed using the Mann–Whitney U test. Categorical variables were presented as frequency (%) and analyzed using either Pearson chi-square test or Fisher’s exact test. Binary logistic regression was employed to analyze the factors influencing postoperative kinesiophobia in patients. A significance level of *α* = 0.05 was used, with *p* < 0.05 considered statistically significant.

## Results

Between May 2023 and January 2024, patients who met the predefined inclusion and exclusion criteria were consecutively enrolled in the study, with prospective collection of their postoperative information. During this period, a total of 1,022 patients were admitted to our hospital for spinal diseases. Among them, we initially enrolled 193 patients with lumbar interbody fusion (LIF), but excluded 12 patients who had undergone MRI at other hospitals and 3 patients who experienced postoperative cerebrospinal fluid leakage and subsequently withdrew from the study. Ultimately, a total of 178 eligible patients were included in the analysis. The demographic characteristics, clinical profiles, and imaging parameters of these enrolled participants are presented in Table 1.

**Table 1:** Fundamental demographic, clinical characteristics, and imaging parameters of the enrolled patients.

Variable	Value	*n* (%)/( $\bar{X}$ ±S)/M (*P*25, *P*75)
Gender	Male	104 (58.4)
	Female	74 (41.6)
Age (YO)		59.629 ± 10.723
BMI (kg/m^2^)		24.234 ± 3.022
Marriage status	Married	168 (94.4)
	Unmarried	4 (2.2)
	Divorced	6 (3.4)
Care provider	Spouse	84 (47.2)
	Children	84 (47.2)
	Others	10 (5.6)
Education level	Junior high school and below	106 (59.6)
	High school	39 (21.9)
	College	27 (15.2)
	Bachelor	6 (3.4)
Job category	None	68 (38.2)
	Primary mental work	30 (16.9)
	Primary physic work	80 (44.9)
Preoperative smoking history	No	133 (74.7)
	Yes	45 (25.3)
Postoperative smoking history	No	156 (87.6)
	Yes	22 (12.4)
Preoperative drinking history	No	160 (89.9)
	Yes	18 (10.1)
Postoperative drinking history	No	172 (96.6)
	Yes	6 (3.4)
LDH with lumbar spinal stenosis	No	30 (16.9)
	Yes	148 (83.1)
Side	Left	63 (35.4)
	Right	68 (38.2)
	Bilateral	47 (26.4)
Disease duration		10.00 (3.00,36.00)
Operative level		2.494 ± 0.928
Postoperation	Within 1wk	14 (7.9)
	1 ~ 4 wks	82 (46.1)
	1 ~ 3 mons	76 (42.7)
	3 ~ 6 mons	6 (3.4)
Number of drainage tubes		1.882 ± 0.490
Total amount of wound drainage (mL)		504.933 ± 223.790
Usage of painkiller pills	None	55 (30.9)
	NSAIDs	110 (61.8)
	Pregabalin	1 (0.6)
	NSAIDs + Pregabalin	9 (5.1)
	Opiates	3 (1.7)
Diabetes	No	145 (81.5)
	Yes	33 (18.5)
CHD	No	165 (92.7)
	Yes	13 (7.3)
Hypertension	No	117 (65.7)
	Yes	61 (34.3)
COPD	No	169 (94.9)
	Yes	9 (5.1)
Arthritis	No	174 (97.8)
	Yes	4 (2.2)
Hepatitis	No	173 (97.2)
	Yes	5 (2.8)
CCI score		2.00 (1.00,3.00)
Exercise with pain	Yes	62 (34.8)
	No	116 (65.2)
Onset time of pain after exercise (min)		30.00 (10.00,30.00)
ODI score		16.00 (12.00,24.00)
VAS score		2.00 (1.00,3.00)
GSES score		24.236 ± 6.024
BDI score		3.00 (1.00,8.00)
Grading of lumbar nerve root compromise		1.972 ± 1.132
ES + MF area (mm^2^)		3696.380 (3153.340,4131.400)

### Incidence and influencing factors of kinesiophobia after LIF

Among the 178 elderly patients, a total of 116 individuals were screened for TSK-17 scores ≥37 points, revealing that motor phobia was present in 65.2% of these patients following LIF (Table 1). The results from univariate analysis demonstrated significant differences between the kinesiophobia group and the non-kinesiophobia group in terms of age, number of surgical levels, postoperative drainage volume, VAS score, GSES score, BDI score, ODI score, degree of nerve root compression and total ES + MF area (*p* < 0.05) (Table 2).

**Table 2:** Association between Tampa Scale of Kinesiophobia index score and related factors.

Variable	Value	TSK < 37 (*n* = 62)	TSK ≥ 37 (*n* = 116)	χ^2^/t/Z	*p* value
Gender				3.399^*^	0.065
	Male	42 (67.7)	62 (53.4)		
	Female	20 (32.3)	54 (46.6)		
Age (YO)		51.452 ± 11.219	64.000 ± 7.409	−7.931^#^	0.000
BMI (kg/m^2^)		24.404 ± 3.338	24.144 ± 2.850	0.547^#^	0.585
Marriage status				3.722^*^	0.155
	Married	58 (93.5)	110 (94.8)		
	Unmarried	3 (4.8)	1 (0.9)		
	Divorced	1 (1.6)	5 (4.3)		
Care provider				3.217^*^	0.200
	Spouse	26 (41.9)	58 (50.0)		
	Children	30 (48.4)	54 (46.6)		
	Others	6 (9.7)	4 (3.4)		
Education level				4.492^*^	0.213
	Junior high school and below	39 (62.9)	67 (57.8)		
	High school	15 (24.2)	24 (20.7)		
	College	5 (8.1)	22 (19.0)		
	Bachelor	3 (4.8)	3 (2.6)		
Job category				4.287^*^	0.117
	None	21 (33.9)	47 (40.5)		
	Primary mental work	7 (11.3)	23 (19.8)		
	Primary physic work	34 (54.8)	46 (39.7)		
Preoperative smoking history				0.060^*^	0.807
	No	47 (75.8)	86 (74.1)		
	Yes	15 (24.2)	30 (25.9)		
Postoperative smoking history				0.100^*^	0.751
	No	55 (88.7)	101 (87.1)		
	Yes	7 (11.3)	15 (12.9)		
Preoperative drinking history				2.911^*^	0.088
	No	59 (95.2)	101 (87.1)		
	Yes	3 (4.8)	15 (12.9)		
Postoperative drinking history				1.921^*^	0.166
	No	62 (100.0)	110 (94.8)		
	Yes	0 (0.0)	6 (5.2)		
LDH with lumbar spinal stenosis				0.425^*^	0.515
	No	12 (19.4)	18 (15.5)		
	Yes	50 (80.6)	98 (84.5)		
Side				0.121^*^	0.941
	Left	23 (37.1)	40 (34.5)		
	Right	23 (37.1)	45 (38.8)		
	Bilateral	16 (25.8)	31 (26.7)		
Disease duration		7.00 (3.00,24.00)	10.00 (3.00,46.00)	−0.496^Δ^	0.620
Operative level		2.097 ± 0.740	2.707 ± 0.951	−4.730^#^	0.000
Postoperation				6.010^*^	0.111
	Within 1wk	2 (3.2)	12 (10.3)		
	1 ~ 4 wks	25 (40.3)	57 (49.1)		
	1 ~ 3 mons	32 (51.6)	44 (37.9)		
	3 ~ 6 mons	3 (4.8)	3 (2.6)		
Number of drainage tubes		1.952 ± 0.493	1.845 ± 0.486	1.388^#^	0.167
Total amount of wound drainage (mL)		367.790 ± 177.446	578.233 ± 211.710	−6.672^#^	0.000
Usage of painkiller pills				2.437^*^	0.752
	None	19 (30.6)	36 (31.0)		
	NSAIDs	41 (66.1)	69 (59.5)		
	Pregabalin	0 (0.0)	1 (0.9)		
	NSAIDs + Pregabalin	2 (3.2)	7 (6.0)		
	Opiates	0 (0.0)	3 (2.6)		
Diabetes				3.310^*^	0.069
	No	55 (88.7)	90 (77.6)		
	Yes	7 (11.3)	26 (22.4)		
CHD				0.345^*^	0.557
	No	56 (90.3)	109 (94.0)		
	Yes	6 (9.7)	7 (6.0)		
Hypertension				1.159^*^	0.282
	No	44 (71.0)	73 (62.9)		
	Yes	18 (29.0)	43 (37.1)		
COPD				0.208^*^	0.649
	No	60 (96.8)	109 (94.0)		
	Yes	2 (3.2)	7 (6.0)		
Arthritis				0.000^*^	1.000
	No	61 (98.4)	113 (97.4)		
	Yes	1 (1.6)	3 (2.6)		
Hepatitis				0.000^*^	1.000
	No	60 (96.8)	113 (97.4)		
	Yes	2 (3.2)	3 (2.6)		
CCI score		2.00 (1.00,3.00)	2.00 (0.00,3.00)	−0.191^Δ^	0.848
Exercise with Pain				0.735^*^	0.391
	Yes	19 (30.6)	43 (37.1)		
	No	43 (69.4)	73 (62.9)		
Onset time of pain after exercise (min)		25.00 (10.00,30.00)	30.00 (10.00,30.00)	−1.535^Δ^	0.125
ODI score		15.00 (11.00,18.00)	17.00 (13.00,27.50)	−2.412^Δ^	0.016
VAS score		1.00 (0.00,2.00)	3.00 (2.00,3.00)	−7.389^Δ^	0.000
GSES score		27.919 ± 5.695	22.267 ± 5.236	6.654^#^	0.000
BDI score		2.00 (0.00,4.00)	5.00 (2.00,9.00)	−4.140^Δ^	0.000
Grade of lumbar nerve root compromise		1.19 ± 1.053	1.62 ± 1.027	−2.620^#^	0.010
ES + MF area (mm^2^)		3846.38 (3453.34,4173.18)	3648.34 (3071.92,4076.98)	−2.513^Δ^	0.012

*Chi-square test, #: t-test, ^Δ^: Z test.

In order to further investigate the correlation between the 9 variables exhibiting single factor differences and the occurrence of kinesiophobia, as well as to explore their relationship, we employed a binary logistic regression model (Table 3). The occurrence of kinesiophobia was considered as the dependent variable, while the 9 variables with single factor differences were treated as independent variables. All data were standardized prior to conducting binary logistic regression analysis.

**Table 3:** Influencing factors of kinesiophobia in patients after LIF: binary logistic regression analysis.

Variable	B	SE	Wald	OR	95%CI		*p*
					Lower limit	Upper limit	
Age (YO)	1.357	0.428	10.044	3.883	1.678	8.985	0.002
Operative level	0.745	0.351	4.495	2.106	1.058	4.194	0.034
Total amount of wound drainage (mL)	1.315	0.519	6.423	3.724	1.347	10.294	0.011
VAS score	1.482	0.484	9.389	4.401	1.706	11.357	0.002
GSES score	−1.107	0.456	5.908	0.330	0.135	0.807	0.015
BDI score	1.379	0.600	5.283	3.969	1.225	12.859	0.022
ODI score	−0.446	0.0393	1.286	0.640	0.296	1.384	0.257
Grade of lumbar nerve root compromise	1.593	0.397	16.072	4.916	2.257	10.710	*P*<0.001
ES + MF area (mm^2^)	−0.678	0.338	4.037	0.508	0.262	0.984	0.045
Constant	2.206	0.494	19.947	0.000			9.075

B, Beta; SE, Standard Error; OR, Odds Ratio; CI, Confidence Interval. R^2^ = 0.596, Adjusted R^2^ = 0.824, *p* < 0.001.

The binary logistic regression analysis revealed that advanced age (OR = 3.883 (1.678–8.985), *p* = 0.002), higher VAS score (OR = 4.401 (1.706–11.357), *p* = 0.002), elevated BDI score (OR = 3.969 (1.225–12.859), *p* = 0.022), reduced GSES score (OR = 0.330 (0.135–0.807), *p* = 0.015), increased number of operative levels (OR = 2.106 (1.058–4.194), *p* = 0.034) and increased postoperative incision drainage (OR = 3.724 (1.374–10.294), *p* = 0.011), greater degree of nerve root compression on preoperative lumbar MRI (OR = 4.916 (2.257–10.710), *p*<0.001), and smaller lumbar paravertebral muscle area (OR=0.508 (0.262-0.984), *p* = 0.045) were independent risk factors for postoperative motor phobia. However, Oswestry Disability Index (OID)score was not an independent factor affecting postoperative kinesiophobia (*p* > 0.05).

## Discussion

Lumbar disc herniation is a degenerative disease of the lumbar spine, characterized by low back pain (LBP) as the primary symptom, and it is a prevalent clinical condition in orthopedics. This condition primarily affects individuals between 25 to 55 years old, with a higher incidence among males than females. Approximately 10 to 20% of patients require surgical intervention (30), and postoperative exercise rehabilitation plays a crucial role in facilitating early return to work. However, fear and avoidance behaviors toward exercise after surgery hinder compliance with rehabilitation exercises, impede the recovery of lower back muscles (31), and fail to alleviate residual pain symptoms postoperatively, leading to prolonged impairment of motor function and perpetuating a vicious cycle (31). In this study, motor phobia was observed in 65.2% of patients following lumbar interbody fusion (LIF), which aligns closely with findings reported by Kemani et al. (31) but slightly exceeds those reported by Svensson et al. (32) and Lv et al. (33) for simple lumbar discectomy and elderly patients with primary osteoporosis, respectively. Notably, the prevalence of kinesiophobia among patients with chronic heart failure or chronic obstructive pulmonary disease surpasses that reported by Fatih et al. (34). The rationale behind this discrepancy may be attributed to LIF being more invasive compared to simple lumbar discectomy; furthermore, patients undergoing LIF may possess an increased desire for self-protection when compared to non-surgical counterparts due to perceiving safety as paramount during physical activities given the high-risk nature associated with LIF surgery (35).

The incidence of kinesiophobia varies across different countries, regions, surgical procedures, and disease types. This study reveals a high prevalence of kinesiophobia among patients after lumbar interbody fusion (LIF) in our region. Given the increasing age of individuals with lumbar disc herniation and the aging population, it is crucial to recognize the significant detrimental effects of kinesiophobia. The objective of this study was to investigate the incidence of kinesiophobia in LIF patients and identify associated risk factors to gain further insights into its underlying mechanisms. Our findings indicate that advanced age, higher visual analog scale (VAS) scores for pain intensity, elevated Beck Depression Inventory (BDI) scores, increased General Self-Efficacy Scale (GSES) scores, multiple operative segments involved, greater postoperative incision drainage volume, reduced paravertebral muscle area size, and heightened preoperative nerve root compression levels are all independent risk factors for postoperative motor phobia in LIF patients.

The present study revealed a positive correlation between age and the incidence of motor phobia following LIF, which aligns with Alpalha et al.’s findings (36) that physically frail patients and the elderly, particularly those residing in nursing homes, exhibit higher levels of kinesiophobia. Furthermore, previous studies (34) have demonstrated that age significantly influences kinesiophobia levels in patients with chronic heart failure (44.3%) and chronic obstructive pulmonary disease (47.7%). Jenevi et al. (16) observed that individuals aged 56–65 years displayed higher levels of kinesiophobia compared to other age groups, while those over 65 years exhibited even greater levels. Additionally, advanced age may be associated with reduced cognitive function and health information processing abilities (37). Bilgin et al.’s research (26), involving a questionnaire survey on 504 non-surgical patients suffering from neck and low back pain, indicated that individuals with lower educational attainment tend to experience heightened kinesiophobia. However, our study did not identify education level as an independent risk factor for kinesiophobia among LIF patients; furthermore, no significant differences were found in the incidence of post-LIF kinesiophobia across different education levels. These results are consistent with John et al.’s findings (16), suggesting variations in health education experiences related to lumbar spine conditions among subjects with differing educational backgrounds. Notably, individuals with lower educational attainment may benefit from systematic exercise programs and pain self-management health education training conducted by medical professionals (including redefining pain physical examination procedures, scientifically assessing pain severity levels, implementing appropriate pain response measures tailored to individual needs) leading to a reduction in their overall level of kinesiophobia (38).

In this study, there was a positive correlation between the severity of depression (as measured by BDI) and the prevalence of exercise fear following lumbar interbody fusion (LIF), which aligns with findings reported by Bilgin et al. (39). Patients with severe lumbar disc herniation often experience prolonged low back pain, leading to reduced engagement in daily activities and social interactions. Consequently, they develop apprehension toward postoperative exercise rehabilitation due to concerns about potential harm to their bodies. Moreover, depression can intensify pain perception, thereby contributing to the development of exercise phobia associated with pain. Notably, individual self-efficacy emerged as a protective factor against kinesiophobia in our study. Self-efficacy refers to an individual’s confidence in their ability to achieve desired outcomes and diminished self-efficacy has been identified as a predictor for long-term disability (40). Previous research has confirmed that self-efficacy acts as a mediating variable between pain-related fear and avoidance behavior among patients with chronic low back pain (41). Individuals with high levels of self-efficacy exhibit greater activity levels, work endurance, efficiency in exercising/stretching routines, and coping strategies. Furthermore, exercise fear and avoidance can impede individuals’ participation in meaningful activities while increasing negative affective states and reducing self-efficacy levels; ultimately exacerbating pain symptoms and perpetuating a vicious cycle.

Pain is a significant influential factor in the development of kinesiophobia (11), and this study’s findings align with this notion. However, our study revealed that the presence and timing of pain during exercise were not independent factors contributing to kinesiophobia; rather, it was the intensity of pain that emerged as an independent determinant. This implies that individuals experiencing higher levels of pain exhibit greater fear toward engaging in physical activity, with such fear stemming from heightened levels of pain-related apprehension. Previous research has indicated that even after successful surgical interventions, 10 to 40% of patients continue to report persistent pain complaints (37). The persistence of postoperative residual pain can be attributed to various factors including preoperative physiological and anatomical damage severity, surgical variables, as well as psychological and social influences. The fear-avoidance model (42) elucidates the underlying psychological mechanisms whereby individuals with elevated levels of pain-related fear demonstrate biased attentional and cognitive evaluations toward their discomfort, thereby perpetuating both their experience of pain and functional impairments. Notably, individuals exhibiting high levels of pain-related fear exhibit increased activation within the anterior insula and central cingulate cortex when evaluating painful stimuli as potential threats.

Therefore, the intensity of pain stimulus is perceived as heightened, leading to increased vigilance toward pain signals in both internal and external environments. Even after physiological healing has occurred, individuals continue to interpret pain as indicative of tissue damage progression. However, it should be noted that fear associated with pain does not always result in avoidance behavior; patients experiencing mild to moderate pain may modify their behavior based on self-determined goals. When individuals perceive the value of engaging in target behaviors to outweigh the importance of avoiding pain, they reject avoidance behavior and prioritize participation in exercise training for rehabilitation (43).

In this study, the majority of subjects underwent surgery between 1 and 12 weeks postoperatively, during which time they had their wound drainage tubes removed and were able to ambulate. The patients’ wound healing varied over time, despite following rehabilitation exercise instructions tailored to their specific postoperative period with gradually increasing difficulty. However, patients who were closer to the operation date still expressed concerns about poor wound healing and bleeding caused by exercise. Previous studies have indicated a strong correlation between increased placement of drainage tubes after surgery and higher rates of surgical site infection (44). Additionally, greater incision drainage following surgery may indicate more vascular injury, impaired coagulation function, and lower hemoglobin levels in patients (45). Consequently, such patients tend to be more cautious after surgery as they associate exercise with potential harm or injury and may therefore avoid it altogether. It is important to note that inappropriate exercises like weight lifting, twisting movements, rugby or other contact sports pose certain risks for screw displacement as well as recurrent disc herniation or rupture after lumbar surgery (46). Patients undergoing multiple surgical segments with several postoperative incisions require guidance from professional rehabilitation therapists for safe exercise practices. This approach not only helps alleviate fear associated with exercising but also prevents unnecessary sports-related injuries.

However, in certain individuals, the recovery of pathological anatomical features in the lumbar spine does not necessarily exhibit a direct correlation with a reduction in pain complaints. This is exemplified by “Failed back surgery syndrome” (FBSS), which denotes persistent axial or peripheral pain following anatomically successful surgical procedures (47). Although LIF can alleviate compression of the intervertebral disc on the nerve root within the spinal canal, it may not instantaneously eradicate nerve root symptoms. A retrospective analysis conducted by Jonsson et al. (11) revealed that 40% of patients experienced enduring back or leg pain after undergoing lumbar disc surgery (48), thereby partially impeding their active movement behavior.

The paravertebral muscle area at the L4 and L5 levels exhibited a significant association with kinesiophobia, as numerous studies have demonstrated that low back pain (LBP) is linked to alterations in paravertebral muscles. A smaller paravertebral muscle area corresponds to weaker lumbar and back muscle strength, which is a crucial factor contributing to low back pain, lumbar degeneration, and dysfunction (49, 50). In this study, we specifically focused on the lumbar core muscles and aimed to establish a direct correlation between paravertebral muscles and exercise phobia. Our findings revealed that both total areas of the paravertebral muscles acted as protective factors against postoperative kinesiophobia. This observation further supports the relationship between the lumbar motor core muscle area and kinesiophobia. Additionally, research has indicated that atrophy of the paraspinal muscles can lead to altered biomechanical properties and lumbar instability, potentially representing the pathological mechanism underlying impaired motor function in patients with lumbar disc herniation (LDH). Furthermore, surgical treatment itself may reduce the cross-sectional area of paraspinal muscles resulting in weakness (51) and stiffness (52).

Additionally, low back pain resulting from the compression of nerve roots in the lumbar intervertebral disc’s nucleus pulposus is a prevalent manifestation among typical patients with lumbar disc herniation (LDH) (53). This condition significantly restricts their engagement in meaningful daily activities prior to surgery. These pain and movement disorders are typically chronic in nature. Although most radicular pain is immediately relieved after surgery, patients may require more time to adapt due to sudden changes in their movement habits post-surgery. Therefore, for the rehabilitation treatment of LIF patients, it may be necessary to implement multimodal rehabilitation measures such as cognitive-behavioral intervention therapy, exercise rehabilitation, and case management (54, 55) to alleviate postoperative pain and fear while facilitating an early return to work.

This study has several inherent limitations. Firstly, this study only included patients with lumbar disc herniation (LDH) from a single center, which may introduce selection bias and limit generalizability to other populations. Secondly, we did not investigate the exercise environment of the included patients, which could potentially act as a confounding factor. Lastly, due to the short follow-up period in this study, it is not representative of exercise fear experienced by patients 1–2 years or longer after lumbar interbody fusion (LIF) surgery nor does it capture dynamic changes in exercise fear at different time points post-surgery. Despite these limitations, this study holds clinical relevance as it provides real-world observations on post-LIF patients in China. In future research endeavors, multi-center studies with larger sample sizes are warranted along with cohort or case–control studies exploring psychological and physiological factors.

## Conclusion

In conclusion, our findings indicate that motor phobia is present in 65.2% of patients with lumbar disc herniation (LDH) following lumbar interbody fusion (LIF). Furthermore, the results of multivariate analysis reveal that advanced age, greater pain severity, higher scores on the Beck Depression Inventory (BDI), lower scores on the General Self-Efficacy Scale (GSES), increased number of surgical levels, higher incidence of postoperative incision drainage, elevated transverse nerve root compression observed on preoperative lumbar magnetic resonance imaging (MRI), and reduced area of the lumbar paravertebral muscles are independently associated with motor phobia. To enhance postoperative functional recovery in LDH patients undergoing LIF and reduce disability rates while improving their quality of life, early identification of individuals at high risk for kinesiophobia is crucial. Implementing interventions such as cognitive-behavioral therapy, exercise rehabilitation management, and preoperative adaptive exercise training should be considered.

## Data Availability

The original contributions presented in the study are included in the article/supplementary material, further inquiries can be directed to the corresponding authors.
